# Advancing personalised and precision nutrition

**DOI:** 10.1017/jns.2026.10084

**Published:** 2026-07-09

**Authors:** Omar Ramos-Lopez, John C. Mathers, Baukje de Roos, María Elizabeth Tejero Barrera, Marie Löf, Sarah E.E. Berry, J. Alfredo Martinez

**Affiliations:** 1 UABC, Mexico; 2 IUNS Precision Nutrition Task Force, UK; 3 Human Nutrition & Exercise Research Centre, Population Health Sciences Institute, University of Newcastle, UK; 4 Rowett Inst. of Nutrition & Health, University of Aberdeen, UK; 5 Lab. of Nutrigenomics and Nutrigenetics, National Inst. of Genomic Medicine, Mexico; 6 Dept. of Biosciences and Nutrition, Karolinska Inst., Sweden; 7 Dept. of Nutrition and Dietetics, Kings College London, UK; 8 IMDEA-Nutrition and CIBERobn, Spain

**Keywords:** Artificial intelligence, Biomarkers, Dietary guidelines, Personalised nutrition, Precision nutrition, Public health

## Abstract

This document presents some advances in personalised and precision nutrition that were addressed in the IUNS-ICN 2025 Congress at August 2025 including the role of biomarkers in personalised and precision nutrition research, personalised and precision nutrition approaches to routine health care services, implications for public health, personalised and precision nutrition around the world, machine learning and precision nutrition, and personalised dietary guidelines alongside population-based policies.

## Introduction

Personalised nutrition typically refers to the use of individual characteristics such as age, sex, diet, and clinical, metabolic, and health traits to design tailored nutritional support for human wellbeing.^(1)^ In this regard, stratified nutrition is a practical approach to personalised diets, grouping people by shared traits like age, gender, lifestyle, or health to offer scalable, targeted advice, bridging generic guidelines and complex individual plans to tailor diets for specific conditions.^(2)^ In turn, precision nutrition adopts a more dynamic, holistic, and multifactorial scope using omics technologies (genomics, epigenomics, transcriptomics, metagenomics, metabolomics), together with socioeconomic and psychological features and food environments.^(3)^ This integrative approach allows the comprehensive analysis and characterisation of large datasets of biological molecules and environmental information using high-quality analytical techniques and bioinformatics tools.^(4)^ The purpose is to improve the efficacy of preventive and therapeutic strategies to mitigate diseases by tailoring them to specific needs and enforcing behavioural changes. This document aims to presents some advances in personalised and precision nutrition that were addressed in the IUNS-ICN 2025 Congress at August 2025. These insights are contributing to a better understanding of the relationship between nutrition, the exposome, and health as well as better characterise potential targets and molecular mechanisms underlying the development of non-communicable diseases (NCDs), or the identification of susceptible individuals based on genomic backgrounds and environment exposures.

## The role of biomarkers in personalised and precision nutrition

Personalised and precision nutrition represents a paradigm shift from generalised dietary guidelines to individualised interventions where their efficacies are based on individual biological, behavioural, and environmental factors. Central to this approach is the use of biomarkers—objective indicators that can measure dietary intake, exposure to nutrients and diets, and physiological responses to dietary interventions. These biomarkers enable researchers and clinicians to better understand the complex interactions between diet and health, and help identify which factors are the best predictors of a response for a particular health outcome, to a particular dietary intervention, and for whom.^(5)^ However, despite significant advances in biomarker discovery in the last two decades, challenges remain in the validation and application of biomarkers in personalised and precision nutrition research.

Accurate dietary assessment is fundamental for precision nutrition, yet traditional self-reported tools are often prone to bias and misreporting. Biomarkers of food intake offer a promising approach to obtaining objective measures of consumption, and therefore, such biomarkers can significantly improve the way we monitor compliance and reduce error in diet-health association studies. However, the field is still in its infancy and thus far, we only have limited examples to demonstrate an ability to quantify food intake. Currently, a few hundred potential biomarkers of food intake have been identified for less than 100 foods, with robust markers having been developed for whole grains, soy, and sugar. Whilst the focus thus far has been very much on identifying new food intake biomarkers, the validation of such biomarkers has been lagging behind, restricting their application in large-scale studies.^(6)^ Furthermore, there are a number of technical considerations which will need to be addressed in order to move this field forward. An important consideration is the highly dynamic nature of the metabolome, making single measurements often insufficient for an accurate dietary assessment. But repeated sampling to capture the variability in the metabolome is not necessarily done nor practical for most studies. Furthermore, many biomarkers may only reflect short-term dietary intake, whereas the assessment of dietary intakes over longer periods of time, to reflect habitual intake, could be really valuable.^(7)^ Also, food composition databases only cover a very small fraction of all the biochemical compounds that are present in foods, making it difficult to assess which foods contribute to which nutrients and non-nutrients, and their metabolites, in individual metabolomes.^(8)^ To advance this field, research should focus on expanding biochemical coverage of food composition databases but also on validating biomarkers of food intake in more diverse populations. Integration of artificial intelligence (AI) approaches could further enhance dietary intake prediction from metabolomic data.

Variability in individual responses to similar diets presents both a challenge and an opportunity for precision nutrition. Dietary interventions interact with genetics, physiology, the microbiome, underlying health status, behaviours, social influences, and environmental exposures to affect health outcomes such as metabolic responses, biomarkers of chronic disease, biomarkers of ageing, biomarkers of inflammation, and sensory/emotive responses to food.^(5,8)^ This means that understanding the dietary responses of an individual, or between individuals, requires us to understand the complexity of endogenous and exogenous interactions on the genetic, physiological, and environmental level. Exposome-wide association studies highlight the level of this complexity but also the importance of measuring all environmental exposures, in addition to genetics, using accurate and precise biomarkers in a robust manner in order to quantify their contributions to a clinically relevant outcome. A recent study quantified the relative contributions of a large range of environmental exposures and genetics on ageing outcomes and premature mortality and found that polygenic risk scores explained less than 2%, whilst environmental exposures accounted for 17%, of mortality variation.^(9)^ Interestingly, across all environmental exposures, diet contributed minimally to ageing outcomes, likely due to poor dietary quantification in the Biobank cohort that was being used for the data analysis. These findings underscore the need for improved biomarkers of dietary intake, and in addition, the inclusion of biomarkers that reflect multiple contextual factors like psychosocial and environmental determinants, including stress, social support, and financial stability. These contextual factors are mostly ignored in physiological studies that are focusing on cardiometabolic outcomes such as postprandial glucose metabolism. As this outcome can be monitored continuously, it is often used as a primary outcome in precision nutrition studies.^(10,11)^ But what is often not considered is that behavioural and environmental contextual factors can contribute significantly to related health outcomes, like weight loss. Indeed, predicting weight loss based on algorithms developed to optimise postprandial glucose metabolism alone may not be adequate and effective.^(12,13)^

Future research should therefore prioritise relevant exposure and outcome biomarkers that can be measured continuously, enabling the dynamic monitoring of health and environmental trajectories, where the integration of exposome data with dietary biomarkers could provide a more holistic understanding of nutrition’s role in health and disease. *N*-of-1 trials—where repeated measures of exposure and outcome biomarkers within individuals replace traditional group comparisons—offer a powerful approach to assess responsiveness to interventions.^(14,15)^ *N*-of-1 designs provide statistical power to detect individual-level effects, enabling the identification of exposure biomarkers that significantly and independently affect an outcome on the individual level, facilitating personalised recommendations. For high-granular data, we will increasingly need modelling and AI approaches and digital twins are an exciting new development in personalised nutrition. Recently this approach was used to predict a diet response before and after long-term fasting as an intervention. The development of an offline mathematical model was able to describe mechanisms regulating diet response and fasting metabolic fluxes, with the tool being able to mechanistically explain and integrate data from several clinical studies and correctly predict new independent data, even those that could not be measured in vivo, such as hepatic rates of gluconeogenesis, in response to fasting and different diets. Such models are particularly valuable if metabolic responses can be successfully adapted to a specific individual’s sex, weight, and height, as well as to an individual’s historical data on metabolite dynamics.^(16)^

In conclusion, biomarkers are indispensable for advancing personalised and precision nutrition, yet their development and validation remain incomplete. Robust biomarkers of food intake can transform dietary assessment, while a larger selection of exposure and response biomarkers, measured dynamically, will enable a more nuanced and mechanistic understanding of diet-health interactions on the individual and population level. Emerging methodologies, like *N*-of-1 trials, deep phenotyping, and AI-driven modelling, combined with enhanced data infrastructure and rigorous validation to ensure biomarkers are reliable, scalable, and clinically and environmentally relevant, show promise to address some of the current limitations, paving the way for the development of more individualised nutrition strategies.

## Taking personalised and precision nutrition approaches to routine health care services

Personalised and precision nutrition is a real game-changer with staggering potential to improve dietary interventions and moving away from the current one-size-fits-all approaches for preventing and managing NCDs.^(17)^ Extensive research initiatives are being spent on identifying disease risk prediction by a combination of multidimensional data such as phenotypes, multi-omics, and lifestyle information to guide tailored dietary advice. However, one aspect that has received less attention is the next step, i.e., the translation and implementation of these approaches to routine health care services after their evidence has been shown.^(18)^ Importantly, we can learn from the last two decades of research on digital lifestyle interventions. To illustrate, digital lifestyle interventions have shown promising results to improve dietary and physical activity behaviours, cardiovascular risk factors and self-management.^(19–21)^ However, thus far, relatively few research-based interventions have actually been implemented in routine health services at scale.^(22,23)^ We recently published a comment paper in Nature Medicine where we presented six recommendations for how researchers can accelerate and scale the translation of their digital health interventions into routine practice.^(23)^ Altogether, they can also help researchers to implement their personalised and precision nutrition tools and avoid them from remaining in experimental and academic settings as is currently often the case.^(18)^ Briefly, they include a) more research on the actual implementation is urgently needed; b) implementation should be considered already in the research phase; c) all relevant stakeholders should be engaged in all aspects of the research; d) an appropriate business model for implementation should be established already in the research phase; e) use a future-proof approach; f) ensure inclusiveness and accessibility of the tool for all irrespectively of language and literacy level. The latter is essential; however, inclusiveness is often discussed in the light of the fact that personalised and precision nutrition studies should include diverse and representative populations. This is clearly very important to improve quality of risk prediction models and algorithms; however, we also need more research on how we can adapt personalised and precision nutrition interventions to address different needs of users (e.g., languages and literacy levels) so that they can also be accessed and used by socially disadvantaged populations who have higher prevalence of many NCDs.

## Precision nutrition implications for public health

The need to improve eating patterns globally is both major and urgent. In addition to the adverse impacts of human food production, retail and consumption on climate change and environmental degradation, unhealthy diets are responsible for approximately 15 million avoidable deaths each year^(24)^ and very substantial morbidity due to greater risk of all common NCDs.^(25)^ Recent modelling studies have predicted gains in life expectancy of up to 10 years following sustained change from unhealthy dietary patterns to those associated with greatest longevity.^(26–28)^ Consequently, for more than two decades, action to improve diets has been central to national and global public health strategies.^(29)^ Personalised and precision nutrition approaches have been shown to improve eating behaviour^(30)^ and so have potential to contribute to meeting these public health goals.

However, to date, most personalised and precision nutrition have been delivered to relatively small numbers of people and their cost and complexity create challenges when considering how to scale up for delivery to communities and countries. In addition, most personalised and precision nutrition interventions make substantial demands on participants in respect of both time and input (providing data and biological samples) which means that they are likely to be attractive to more affluent individuals who have the resources to do so and who have interests in health or health technologies.^(31)^ Further, dietary behaviour and health are patterned socioeconomically^(32)^ with poorer people not only dying younger than wealthier people^(33)^ but also living more of those shorter lives with chronic diseases.^(32)^ Consequently, if personalised and precision nutrition interventions are more accessible to, or more readily adopted by, those with greater socioeconomic advantage, improvement in public health overall may be limited and health inequalities may be exacerbated.^(34)^

There is good evidence that improving food environments will make an important contribution to improving dietary intake and, ultimately, diet-related health,^(35)^ and such approaches have the significant advantages that they do not require active engagement by individuals, are likely to have attractive cost: benefit ratios and are likely to benefit most the less socioeconomically advantaged sections of society. However, there has been very slow progress in improving dietary patterns at population levels^(25)^ with limited impact on public health. Consequently, there is an important opportunity for personalised and precision nutrition approaches to complement other strategies for achieving better diets for all. However, achieving this aim will require greater focus on the psychosocial, cultural and economic factors that drive individual dietary behaviours over the long term^(36)^ and, therefore, that influence health and longevity. Such approaches are being advocated in some more recent frameworks,^(37)^ but the emphasis on biological factors remains dominant in most personalised and precision nutrition interventions. In addition, there are important opportunities to combine participatory co-design approaches to develop more culturally appropriate personalised and precision nutrition interventions.^(38)^ Scalability and affordability are likely to be enhanced by developments in AI to facilitate management and interpretation of the complex and dynamic information flows that underpin personalised and precision nutrition interventions that deliver attractive, timely, and actionable advice and support for sustained dietary improvement.^(31)^ If those designing future personalised and precision nutrition interventions prioritise the needs of less advantaged sections of society with the goal of improving health equity, this area of nutrition science has considerable potential to move on from the hype of a new technology and to make a useful contribution to better public health.

## Personalised and precision nutrition in Latin America: challenges and opportunities

The study of the factors that influence the individual response to a treatment, such as a diet, food or nutrient, has been of great interest for the scientific community. Understanding the sources of inter-individual variation is key to develop efficient strategies for prevention and treatment of diseases. One of the first sources of individual variation that was investigated was genetics. The sequencing of the human genome map reveled great variation, and the rapid technological progress of tools for the study of large numbers of variants accelerated research in this area. Over time, numerous studies demonstrated that other factors besides genetic variation contribute to the diversity of responses to diet, which have emerged from the study of the transcriptome, epigenome, proteome, metabolome or other collections of biological molecules.^(39–41)^ In addition, the development of technology such as smartphones, watches, apps and similar, allows for the registration and analysis of data on dietary intake, sleep, physical activity and other components of lifestyle in real time, producing large amounts of data that may be used for research using novel tools and analytical models. However, the application of these analytical methods for clinical and public health purposes requires of further scientific support and are costly and unaffordable for many people, especially in low and middle-income countries.

Nutrition research has been very active in Latin American since the early 20th century due to the presence of malnutrition. Over decades, the epidemiological challenges have changed, and countries face complex health profiles with a high prevalence of obesity-related diseases,^(42,43)^ and some nutritional deficiencies in the less favoured groups. Inequality in access to medical care, among other socio demographic factors influencing health are also present across Latin American countries.

Latin American populations show large genetic and environmental diversity, with variables admixture of Native American, European and African ancestry, and are underrepresented in genetic studies, since only 0.38% of participants in genome wide studies are of Latin American origin. The Genetics of Latin America Diversity database is a recently created platform that provides support for epidemiological studies on these populations.^(44)^ To date, a small number of countries in Latin America have a consistent scientific production on precision nutrition-related topics such as nutrigenetics, nutrigenomics, epigenetics, and the role of gut microbiome in nutrition, using omics methods and bioinformatics.^(42,43)^ Most studies address common diseases such as obesity, type 2 diabetes, and related conditions. Some researchers have also investigated the molecular effects of local and traditional foods, exploring gene-diet interactions and their effects on health.^(45,46)^

Precision nutrition has evolved by the integration of data obtained with advanced technology; however, the development of models using data generated by common analytical methods is possible and may be used for research. In turn, personalised nutrition investigation may be conducted with non-omic data, using available information and technology such as clinical tests to define metabolic signatures and incorporate other relevant individual data. Some countries conduct national surveys on health, producing valuable information for research and decision making, there are also large databases of clinical and epidemiological data obtain by different studies. Tools based on AI can analyse large datasets, uncovering patterns that other methods may overlook. Collaborative networks between institutions across countries sharing expertise and technology may significantly contribute to development of personalised and precision nutrition fields.

Furthermore, efforts such as the Samba,^(47)^ or SoMeMi projects are directed to the study of the gut microbiome in different regions of Latin America. In conclusion, research on personalised and precision nutrition is growing in Latin America with limited funding and technology. Research is required to establish methods to provide patients with improved dietary strategies and to incorporate personalised and precision nutrition-derived strategies into public health.^(48)^ Research on personalised nutrition may be conducted with non-omic data, using available information and technology. However, the contribution of metabolomic, genomic, and metagenomic information is very relevant and having data from underrepresented populations is important. Collaboration is key to improve the current knowledge, and international projects are required to speed up and share research.

## Machine learning and precision nutrition

The interpretation and better understanding of the complex interactions between diet and human metabolism often requires the use of high-performance analytical statistics for efficient handling and results delivery.^(49)^ In this regard, machine learning (ML) and other AI advances are allowing the operative integration and interpretation of multiple data for the diagnosis, prognosis and management of NCDs.^(50)^

ML relies on diverse algorithms or mathematical/logical instructions to learn from a set of data to solve questions or make decisions.^(51)^ In general, ML covers supervised and unsupervised methods, where supervised ML develop tasks for defining categories, groups classification, and regression tasks to predict outcomes, while unsupervised ML comprises exploratory data analysis for clustering and variable dimensionality reduction.^(52)^

ML and AI have improved biomedical data analysis by enabling the identification of complex patterns and nonlinear relationships beyond traditional statistical methods, which facilitate the characterisation of risk profiles, screening of biomarkers, and prediction of personalised responses to treatments or dietary interventions.^(53)^ A comprehensive analysis in precision nutrition research include multi-omics screening and integration data from metabolomics, metagenomics, nutrigenetics, nutrigenomics, epigenomics, and microbiota profiling.^(54)^

In this context, ML algorithms can create models enabling the identification of microbial signatures linked to phenotypical traits (i.e., visceral adiposity) by integrating multiple layers of data, including body composition, lifestyle factors, genetics, and metagenomic profiles.^(55)^ Also, multiple genetic, phenotypic, and exogenous factors predicted weight loss depending on the consumption of hypocaloric diets with different macronutrient composition, which may help to personalise dietary advice for the management of obesity using precision nutrition variables.^(56)^ Furthermore, a ML model was constructed to predict obesity using genome-wide and epigenome-wide gene-gene and gene-diet interactions, thus extending current knowledge of the drivers of obesity and guiding precision nutrition strategies for the prevention and treatment of obesity.^(57)^ ML insights can be also used for grouping of samples and categorisation of nutritypes based on the response to dietary intake or the implementation of nutritional scores.^(58)^

ML tools can be translated into clinical practice including supporting decision-making and development of diet optimisation systems for precision nutrition.^(59)^ However, some limitations affecting ML strategies and implementation include limited generalisability across diverse populations (meaning models may not work effectively for all people), the availability of technical/organisational infrastructures, powerful computer systems, personal critical expertise, and ethical considerations concerning the use of personal information.^(60)^ Some strategies to overcome these limitations include researcher collaborations for the construction and/or combination of large emerging international databases multiple diverse information to compute accurate ML models and externally validate them to ensure generalisability in different populations. Other actions comprise the development of standards for data sharing considering the biological and clinical ecosystems and the creation of smart computational and analytical tools to increase speed and capacity of dealing with data heterogeneity harmonisation (in terms of biological, social and environmental determinants of health) and coherent data grouping.^(59)^ Accordingly, training health professionals with appropriate bioinformatics knowledge to manage, interpret and properly use the information generated by AI is essential as well as supervision for skilled professionals.^(60)^ Furthermore, critical ethical considerations include the protection of sensitive personal data, as the results can have far-reaching implications for the health and legal status of the consumer.

## A place for personalised dietary guidelines alongside population-based guidelines?

Research on personalised nutrition has traditionally focused on individualisation based on complex biological mechanisms and phenotypic features. However, findings from large-scale research programmes, such as the PREDICT programme,^(61)^ alongside challenges in translating approaches into practice, indicate that realising the full potential of personalised nutrition requires a broader conceptualisation of personalisation. Specifically, effective personalised nutrition must incorporate not only biological characteristics but also behavioural, contextual, and environmental factors, including *how* individuals eat, *what* they currently eat, *why* they make particular food choices, their built environment, and their preferred modes of receiving dietary advice. These dimensions of personalisation have long been integral to dietetic practice and are essential for the effective delivery of personalised dietary advice at scale.

Addressing these multiple features of personalisation may enable personalised nutrition to tackle two key challenges: adherence to dietary advice and the efficacy of that advice. Although population-based dietary guidelines are grounded in rigorous scientific evidence, less than 1% of individuals adhere to the core dietary recommendations.^(62)^ Furthermore, there is increasing recognition that individuals respond differently to dietary recommendations compared with the population mean.^(63)^ As a result, certain dietary interventions may be more effective for some individuals than for others. Personalised approaches therefore have the potential to improve both efficacy and adherence,^(64)^ complementing rather than replacing population-based guidance.

For example, in the UK, the Scientific Advisory Committee on Nutrition recommends reducing salt intake to 6 g/day, based on evidence that habitual high salt consumption increases blood pressure and, consequently, the risk of stroke and premature cardiovascular mortality.^(65)^ However, evidence indicates that responses to salt reduction are highly individual and depend in part on biological factors such as plasma renin status. Approximately 75% of individuals experience a reduction in blood pressure following salt restriction to varying degrees, whereas around 25% show no improvement.^(66)^ Similar inter-individual variability has been reported for responses to other nutrients, including saturated fat, dietary cholesterol, and polyphenols.^(67)^ Thus, while population-level reductions in sodium or saturated fat intake are likely to improve overall public health, tailoring dietary advice according to individual responsiveness may enhance both the efficacy of interventions whilst motivating people to adhere to recommendations.

A major challenge for personalised nutrition lies in its practical implementation: how to integrate population-based dietary advice with individualised recommendations at scale while ensuring accessibility and equity. Achieving this requires disentangling the complexity of individual biology, dietary behaviours, lifestyle patterns, and the underlying drivers of food choice. Addressing this challenge necessitates the collection of data at a scale, breadth, depth, and precision that is now achievable through the integration of remote clinical testing, digital technologies, and community science. Moreover, continuous, real-time data collection enables dynamic and adaptive modification of dietary advice over time.

The PREDICT research programme, which has generated multidimensional data from more than 300,000 individuals, exemplifies the potential of big data approaches that leverage novel technologies and community participation. Findings from the PREDICT studies have demonstrated substantial inter-individual variability in postprandial responses to food, largely driven by meal context (e.g. sleep, physical activity, time of day), the gut microbiome, physiological characteristics, and habitual diet, whereas genetic factors explained a relatively small proportion of the observed variability.^(11,68)^ Ongoing analyses have further highlighted the importance of behavioural and lifestyle factors—particularly *how* individuals eat and live—as potentially underexploited targets for personalised nutrition. Factors such as snacking timing, fasting duration, eating speed, meal sequencing, and sleep patterns all influence metabolic responses to food.

Ultimately, the key challenge for the field is the translation of individual-level complexity into improved public health strategies at the population level. A promising solution may lie in combining stratified nutrition approaches within national dietary guidelines with personalised overlays that adapt population-based advice to individual characteristics, behaviours, and contexts.
